# Helminths assemblage in two opossum’s species, *Didelphis albiventris* and *Didelphis aurita* (Mammalia: Didelphimorphia), from the Atlantic Forest of Argentina

**DOI:** 10.1017/S0031182025000563

**Published:** 2025-04

**Authors:** Bárbara Betina Hartmann, Ezequiel Andres Vanderhoeven, Dante Luis Di Nucci, Agustín Solari, Juliana Notarnicola

**Affiliations:** 1 Facultad de Ciencias Forestales, Instituto de Biología Subtropical (IBS), Universidad Nacional de Misiones – CONICET, Laboratorio de Biodiversidad Parasitaria de Animales Silvestres, Puerto Iguazú, Misiones, Argentina; 2 Laboratorio de Diagnostico Vegetal y Animal (LADEVA), Universidad Nacional de Misiones Facultad de Ciencias Forestales, Eldorado, Misiones, Argentina; 3 Centro de Rescate, Rehabilitación y Recría de Fauna Silvestre Güirá Oga, Fundación de Historia Natural Félix de Azara, Puerto Iguazú, Misiones, Argentina

**Keywords:** Atlantic Forest, *Didelphis*, helminth assemblage, Misiones, opossums

## Abstract

In the Argentinian Atlantic Forest (AAF) of Misiones, opossums comprise 13% of the wild mammalian diversity. The white-eared opossum, *Didelphis albiventris*, and the southern black-eared opossum, *D. aurita* are sympatric marsupials, and the most frequent mammals in the northern Misiones. In this study, we describe the helminth assemblages from both *D. albiventris* and *D. aurita* in the northern AAF. We found a total of 15 species of helminths: 2 trematodes, 1 cestode, 11 nematodes and 1 acanthocephalan. The specific richness in *D. albiventris* was 12, while in *D. aurita* was it 13. Both opossum’s species share 10 helminth species; *D. albiventris* presented *Capillaria* sp. 2 and *Globocephalus marsupials*, absent in *D. aurita*; while *D. aurita* presented *Trichuris didelphis, Capillaria* sp. 1, and *Travassostrongylus orloffi*, absent in *D. albiventris. Cruzia tentaculata* registered the highest prevalence in both opossum species. Seven out of the 12 helminth species identified in *D. albiventri*s have an indirect life cycle. Similarly, in *D. aurita*, 5 out of 13 helminth species exhibit an indirect life cycle. This suggests that nearly half of the assemblage of helminth in both opossum species need an intermediate host acquired through the diet. We also present new records for Argentina including *Trichuris minuta, G. marsupialis, Viannaia viannai, T. orloffi* and *T. callis*. This is the first time the helminth assemblage has been described for *D. aurita* in Argentina.

## Graphical abstract

**Figure:**
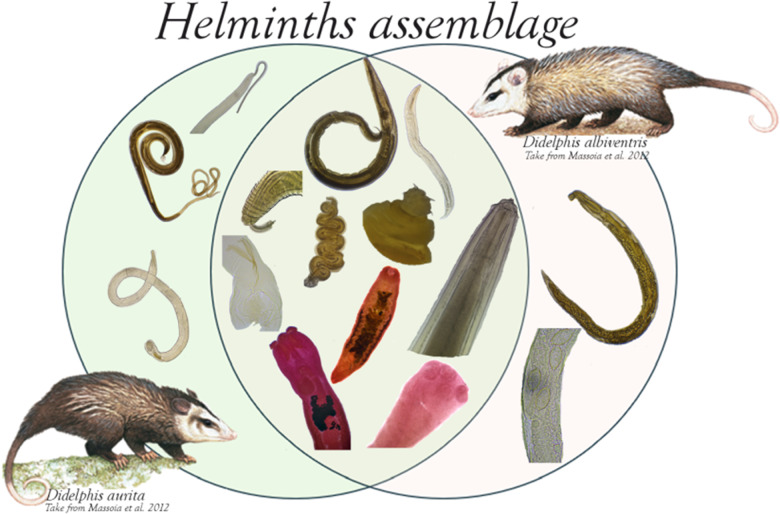

## Introduction

The Atlantic Forest (AF) is widely recognized as one of the world’s top 5 biodiversity hotspots (Myers et al., 2000). The province of Misiones in Argentina, represents the largest continuous remnant, harbouring approximately 30% of the country’s overall biodiversity (Placi and Di Bitetti, 2006).

In Misiones, opossums comprise 13% of the wild mammalian diversity and they are represented by 9 genera and 15 species: *Caluromys* Allen, 1900, *Chironectes* Illiger, 1811, *Didelphis* Linnaeus, 1758, *Gracilinanus* Gardner y Creighton, 1989, *Lutreolina* Thomas, 1910, *Metachirus* Burmeister,1854, *Micoureus* Lesson, 1842, *Monodelphis* Burnett, 1830, and *Philander* Tiedemann, 1808 (Massoia et al., 2012). The white-eared opossum (*Didelphis albiventris* Lund, 1840) and the southern black-eared opossum *D. aurita* Wied-Neuwied, 1826 are the so called medium sized opossums, frequently found in human dwellings and peripheral areas in north Misiones (Massoia et al., 2012).

*Didelphis albiventris* is distributed in Brazil, Bolivia, Paraguay, Uruguay and Argentina (Wilson and Reeder, 2005). In Argentina, this species is distributed from north to the central part of the country (Chemisquy and Gabriel, 2019) with an expanding presence toward the south (Pastrán-López et al., 2022). This opossum is found in diverse habitats like forests and grasslands often associated with human-altered landscapes (Massoia et al., 2012). *Didelphis aurita* is distributed along the coast of Brazil, from Bahia to Rio Grande do Sul, east of the lower Paraguay river and the Argentinian Atlantic Forest (AAF) (Wilson and Reeder, 2005). Unlike *D. albiventris*, it is associated in well-preserved native forests, both in continuous areas and remnants (Massoia et al., 2012; Chemisquy and Gabriel, 2019). Both species are omnivorous and have opportunistic feeding behaviour, with a diet consisting of small vertebrates, invertebrates, seeds and fruits (Massoia et al., 2012). Additionally, these opossums may consume garbage remnants of human consumption, as well as food available inside the forest (Bezerra-Santos et al., 2021).

In Argentina, studies on helminths in opossums are scarce and sporadic over time (Hartmann, 2023). At present, about 27 helminth species are known to be present in the most common species of opossums, mostly from central Argentina (Santa Cruz et al., 1999; Lunaschi and Drago, 2007; Castaño Zubieta et al., 2014; Montes de Oca et al., 2024) and a few records from a rare species in the Northwest Argentina (Navone, 1989; Navone et al., 1991; Navone and Suriano, 1992). To our knowledge, in the AF there are several studies that report helminths in *D. aurita* and *D. albiventris* (see Antunes, 2005; Ramos et al., 2016; Costa-Neto et al., 2018; Bezerra-Santos et al., 2021). However, in Misiones there is only one record of Martínez (1986) mentioning the trematode *Duboisella proloba* Baer, 1938 in *D. albiventris*.

As opossums are frequent in urban and rural environments, live in close relationship with humans and domestic animals, they are considered potential reservoirs of many infectious agents (i.e. *Trypanossoma cruzi, Leishmania infantum, Ancylostoma caninum, Angiostrongylus cantonensis*, among others) (Bezerra-Santos et al., 2021). Several studies have shown that opossums are involved in the transmission of parasites of animal health concern, playing an underestimated role in the epidemiology of parasitic diseases affecting domestic animals (Bezerra-Santos et al., 2021). In this study we proposed to work toward filling this knowledge gap providing new and updated records of helminth species from *D. albiventris* and *D. aurita* in the AAF.

## Materials and methods

### Study site

**Figure 1.**
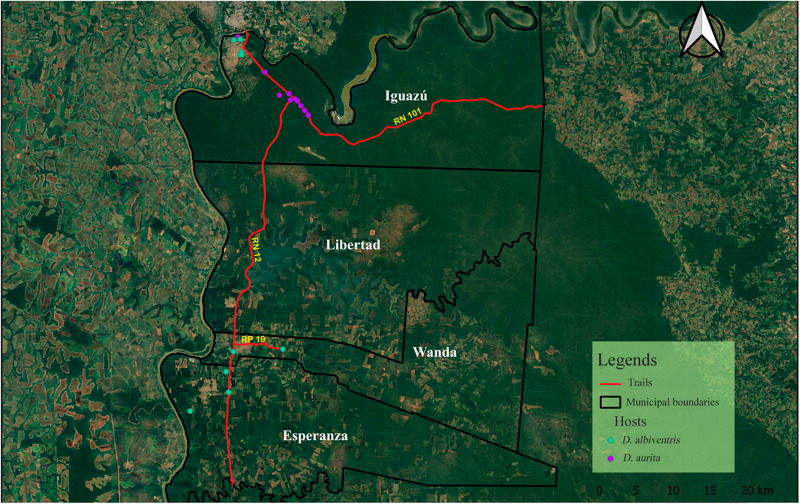
Map of the Iguazú Department. In red is marked the route of National Routes 12 and 101 and Provincial Route 19. Individuals of *Didephis albiventris* collected are marked with green dots and those of *D. aurita* with purple dots.

Our study was conducted between September 2021 and June 2023 in the Department of Puerto Iguazu in the north of Misiones province. Sampling was conducted at various sites, including the routes through Iguazu National Park (National Routes (NR) 12 and 101), Colonia Wanda (NR 12 and Provincial Route (PR) 19), and Puerto Esperanza (NR 12), as well as the urban and rural areas surrounding these cities (Figure 1).

### Sample collection

We collected opportunistically opossum carcasses recently dead, including those from roadkill, dog attacks, or other causes. Specimens were collected in plastic bags and necropsied in the laboratory. The body cavity and viscera were thoroughly examined using a magnifying glass to facilitate observation. We collected and preserved all parasites found. Nematodes and acanthocephalans were fixed in 10% formalin and preserved in 70% ethanol. For morphological studies they were cleared in Amman’s lactophenol and studied with a Leica DM500 microscope equipped with a drawing tube. Trematodes and cestodes were placed between 2 slides with 2% formalin until they were flattened; and preserved in 70% ethanol. For morphological studies specimens to be studied were stained with 1:6 dilutions in 96% ethanol of hydrochloric carmine, dehydrated in a series of alcohol ranging 70%–85%–95%–100% ethanol, rinsed in eugenol, and mounted in Canada balsam. Measurements are given in millimetres (mm) unless other units are stated. Photographs were taken with a Zeiss Primo Star microscope with a compact digital camera.

Voucher specimens were deposited in the Colección de Helmintos Museo de La Plata, Argentina (CHMLP-he) (see Appendix), and IBS authors collection. Host acronyms correspond to the field numbers BH (Barbara Hartmann) and M (Dante Di Nucci), and to the Colección de Mastozoología del Laboratorio de Genética Evolutiva – Dr Claudio Bidau (CM-LGE) from the Instituto de Biología Subtropical IBS (see Appendix).

#### Populations parameters

We analysed parameters at the component population level. Quantitative parameters of prevalence (P), mean abundance (MA) and mean intensity (MI) were calculated following Bush et al. (1997). For the prevalence, a 95% confidence interval (CI) was estimated using Sterne’s method; a bias-corrected and accelerated 95% bootstrap CI (Bca) with 2000 replicates was applied for calculation of MI and MA, according to Reiczigel et al. (2019). The Quantitative Parasitology web software was used to calculate these descriptors (Reiczigel et al., 2019).

## Results

Thirteen specimens of *D. albiventris* and 11 of *D. aurita* were collected. Three species of Platyhelminthes, 10 species of nematodes and 1 species of Acanthocephala were identified. In Table 1 we detail the helminth species and the parameters (P, MI and MA) recovered from both, *D. albiventris* and *D. aurita* in the present study. Below is listed the helminths species including a brief description with their measurements.

**Table 1.** Population parameters of the helminths parasitizing *Didelphis albiventris* and *D. aurita* from Northern Misiones

		*Didelphis albiventris*			*Didelphis aurita*		
Helminths	Infection site	P (%)	MI	MA	P (%)	MI	MA
*Brachylaima migrans*^†^	SI	46.2 (22.4–74)	15 (3–46)	6.9 (1.3–25.8)	54.5 (26.5–80)	25.2 (7.5–57)	13.7 (3.7–40.9)
*Rhopalias coronatus*^†^	SI	15.4 (2.8–43.3)	1*	0.15 (0–0.3)	27.3 (7.9–59.6)	90 (20–142)	24.5 (3.64–74.6)
*Mathevotaenia* sp.^†^	SI	7.7 (0.4–34.2)	438	33.7 (0–101)	9.1 (0.5–40.5)	29*	2.6 (0–7.9)
*Trichuris minuta*	C, LI	53.8 (26–77.6)	14 (1–89)	14.7 (4–37.3)	63.6 (33.3–86.5)	27.1 (12.4–48.6)	17.3 (6.8–37.8)
*Trichuris didelphis*	C	–	–	–	27.3 (7.9–59.6)	4.7 (3–6)	1.3 (0.3–3.2)
*Capillaria* sp.1	S, SI	–	–	–	27.3 (7.9–59.6)	36 (2–68.3)	9.82 (0.5–45.8)
*Capillaria* sp.2	S, SI	15.4 (2.8–43.4)	22 (14–22)	3.4 (0–10.3)	–	–	–
*Globocephalus marsupialis*	SI	7.7 (0.4–34.2)	26*	2 (0–6)	–	–	–
*Viannaia viannai*	SI	23.1 (6.6–52)	15.7 (2–27)	3.62 (0.308–14.6)	72.7 (40.5–92.1)	74.1 (19.6–233)	53.9 (13–205)
*Travassostrongylus orloffi*	SI	–	–	–	9.1 (0.5–40.5)	63*	5.7 (0–17.2)
*Travassostrongylus callis*	SI	15.4 (2.8–43.4)	10.5 (2–19)	1.6 (0–6.2)	18.2 (3.3–50)	49.5 (35–49.5)	9 (0–23.8)
*Aspidodera raillieti*	C, LI	15.4 (2.8–43.4)	5 (4–6)	0.8 (0–2.15)	63.6 (33.3–86.5)	35.6 (9.7–107)	22.6 (5.6–91.3)
*Cruzia tentaculata*^†^	C, LI	92.3 (65.8–99.6)	355 (142–1090)	328 (124–922)	100 (73.6–100)	251 (157–419)	251 (160–413)
*Turgida turgida*^†^	S	30.8 (11.3–58.7)	12 (3–18)	3.7 (0.8–9.4)	45.5 (20–73.6)	5.2 (1.6–9.8)	2.4 (0.6–6.5)
*Oligacanthorhynchus microcephalus*^†^	SI	61.5 (34.2–83.4)	53.9 (20–123)	33.2 (10.5–83.3)	36.4 (13.5–66.7)	8 (2.8–10.9)	2.9 (0.8–6.3)

* ^†^Helminths with heteroxenous life cycle. SI: Small intestine; LI: Large intestine; C: Caecum; S: Stomach; T: Trachea; S: Stomach.

* Only one infected host in the sample, thus CI cannot be calculated.

### Taxonomic aspects

#### Phylum Platyhelminthes Minot, 1876

##### Class Trematoda Rudolphi. 1808

*Superfamily Brachylaimoidea Joyeux and Foley, 1930*

Family Brachylaimidae Joveux and Foley, 1930

**Figure 2.**
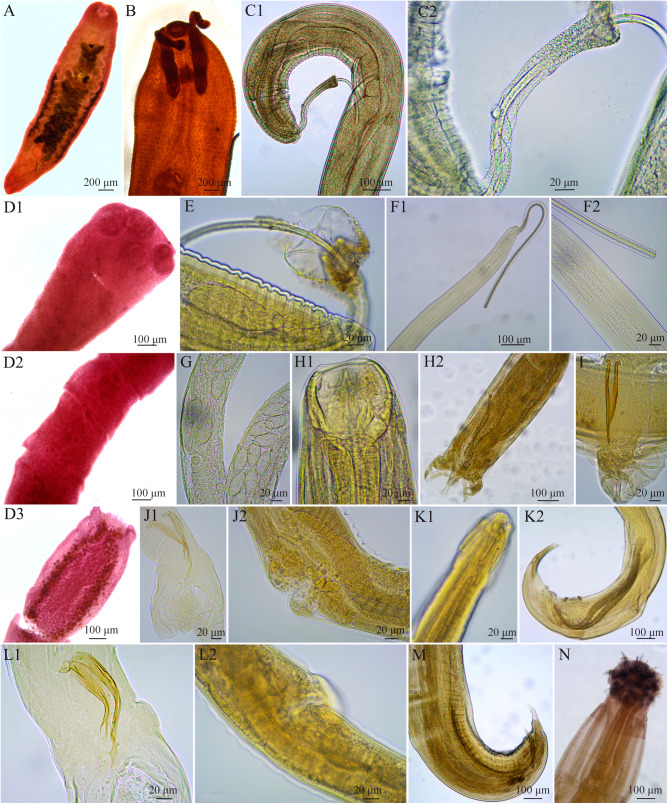
Photographs of helminths: A. *Brachylaima migrans*. B. Anterior end of *Rhopalias coronatus*. C. *Trichuris minuta*: 1. Posterior end. 2. Detail of the spicular sheath. D. *Mathevotaenia* sp: 1. Scolex. 2. Immature proglotids. 3. Mature gravid proglotids. E. Detail of spicular sheath of *Trichuris didelphis*. F. *Capillaria* sp. 1: 1. Posterior end. 2. Detail of spicular sheath and spicule. G. Detail of the vulva and eggs of *Capillaria* sp. 2. H. *Globocephalus marsupialis*: 1. Detail of the anterior region. 2. Tail, detail of bursa and spicule. I. Tail of *Viannaia viannai*. J. *Travassostrongylus orloffi*: 1. Male tail, detail of spicules, gubernaculum, and telamon. 2. Female, detail of the vulva. K. *Aspidodera raillieti*: 1. Anterior region. 2. Tail, detail of spicules, gubernaculum, and sucker. L. *Travassostrongylus callis*: 1. Male tail, detail of spicules, gubernaculum, and telamon. 2. Female vulva with cuticular cap. M. *Cruzia tentaculata*, male tail, detail of spicules and gubernaculum. N. *Oligacanthorhynchus microcephala* proboscis.

*Brachylaima migrans* Dujardin, 1845 (Fig. 2A)

*Description* (n = 5): Trematode small, whitish, elongated subcylindrical body 3.15–4.31 long by 0.60–0.90 wide. Oral and ventral suckers globular, nearly equal, 0.40–0.50 by 0.30–0.60 and 0.30–0.45 by 0.30–0.43, respectively. Oesophagus 0.20–0.30 long by 0.15–0.20 wide. Testes in tandem, located in the posterior third of the body, anterior testis 0.20–0.40 long by 0.20–0.35 wide, posterior testes 0.25–0.45 long by 0.20–0.30 wide. Ovary intertesticular, 0.15–0.25 long by 0.20–0.30 wide, the oviduct 0.2–0.6 long is continued by the uterus 1.75–2.5 long, which open into the genital pore. Eggs elliptical, with one side slightly flattened and the other convex, 0.02 long by 0.01 wide.

Taxonomic summary

*Host: Didelphis albiventris* (BH01, BH06, BH10, M177/22, M190/22, M211/23), *Didelphis aurita* (BH09, BH20, BH24, BH26, BH32, M220/23).

*Infection site*: Small intestine

*Localities*: Puerto Esperanza, Colonia Wanda, and Puerto Iguazú, Misiones, Argentina (see appendix).

*Deposited material*: MLP-He 8141 (1 specimen in a slide).

*Remarks*: The morphological characters are consistent with the description given by Boero and Boehringer (1967). *Brachylaima migrans* has been recorded across Argentina as a parasite of *D. albiventris* and *Lutreolina crassicaudata* Desmarest, 1804 (see Boero and Boehringer, 1967; Martínez, 1986; Santa Cruz, 2006; Lunaschi and Drago, 2007). In Brazil, this species was reported for *D. albiventris* (see Silva and Costa, 1999; Antunes, 2005) and *D. aurita* (see Costa-Neto et al., 2018; Gentile et al., 2022). This is the first record for *D. aurita* in Argentina.

Superfamily Echinostomatoidea Looss, 1899

Family Rhopaliidae Looss, 1899

*Rhopalias coronatus* (Rudolphi, 1819) Stiles and Hassall, 1898 (Fig. 2B)

*Description* (n = 5): Trematodes with elongated, spiny body of 7.35–10.85 long by 1.20–1.50 wide. Specimens possess a pair of armed proboscides with spines, which can be invaginated into a muscular pouch. The pouches open to the exterior on each side of the oral sucker. Subterminal oral sucker. Acetabulum larger than the oral sucker, at 1.40–2.75 from the anterior end. Testes oval, in tandem located in the posterior half of the body, anterior testis 0.50–0.85 long by 0.30–0.60 wide and posterior testes 0.6–0.9 long by 0.3–0.4 wide. Ovary oval, pretesticular, 0.45–0.95 long by 0.4–0.85 wide. Genital pore median, pre-acetabular. Vitelline follicles in lateral fields in posterior third of the body. Eggs oval to elliptical, 0.09–0.1 long by 0.05–0.06.

Taxonomic summary

*Host: Didelphis albiventris* (M190/22, M211/23), *Didelphis aurita* (BH07, BH08, BH20).

*Infection site:* Small intestine

*Locality*: Puerto Iguazú, Misiones, Argentina.

*Deposited material*: MLP-He 8142 (1 specimen on a slide).

*Remarks*: Morphology of our specimens is consistent with the description given by Haverkost and Gardner (2008) and Chero et al. (2017). However, they are larger worms, the tentacle sacs extend far beyond the posterior margin of the pharynx, and the tentacle spines and oral spines are difficult to observe compared to those reported by Haverkost and Gardner (2008). Species of *Rhopalias* are parasites of the small intestine of marsupials from the Nearctic and Neotropical region (Haverkost and Gardner, 2008). *Rhopalias coronatus* has been reported in *D. albiventris* and *L. crassicaudata* from Argentina (see Boero and Boehringer, 1967; Lombardero and Moriena, 1973; Martínez et al., 1973; Martínez, 1986; Haverkost and Gardner, 2008; Jiménez et al., 2024). It was also reported from *D. albiventris* from Brazil and Paraguay (see Haverkost and Gardner, 2008; Marinho de Quadros et al., 2016; Zabott et al., 2017; Teodoro et al., 2019; Jiménez et al., 2024); *D. aurita* (see Costa-Neto et al., 2018; Gentile et al., 2022) from Brazil, and *Philander opossum* Linnaeus, 1758 from Bolivia (Haverkost and Gardner, 2008; Jiménez et al., 2024). This is the first record for *D. aurita* in Argentina.

##### Class Cestoda Rudolphi 1808

*Orden Cyclophyllidea van Beneden in Braun, 1900*

Family Anoplocephalidae Blanchard, 1891

*Mathevotaenia* sp. (Fig. 2D1-D3)

*Description* (n = 5): Small cestodes, 4.95–5.60 in total length, consisting of 19–25 proglottids. Maximum width 0.35–0.45 attained in gravid segments. Scolex unarmed, poorly demarcated from strobila, 0.25–0.30 long by 0.40–0.50 wide. Oval suckers, with thin muscular walls, 0.15–0.2 long by 0.15–0.2 wide. In the specimens studied herein, segmentation begins immediately after scolex. Proglottids trapezoidal with laterally expanded posterior edges. Mature eggs concentrated along the lateral margin of the proglottids. Egg capsule 0.2 by 0.2.

Taxonomic summary

*Hot: D. albiventris* (M211/23) and *D. aurita* (BH20).

*Infection site*: Small intestine.

*Locality*: Puerto Iguazú, Misiones, Argentina.

*Deposited material*: Specimens under study.

*Remarks*: The characteristics of these cestodes correspond to those mentioned by Campbell et al. (2003) for *Mathevotaenia* spp.: small cestodes, craspedote proglottids, scolex unarmed. However, the worms studied herein are smaller than those mentioned by Campbell et al. (2003). *Mathevotaenia argentinensis* (Campbell et al., 2003) and *M. sanmartini* Jimenez, Cambell & Gardner, 2008 present 135–163 proglottids and more than 200, respectively, while *M. bivittata* (Janicki, 1904) has 37–49 proglottids. Our specimens have fewer proglottids, and the position of the ovary and testes differs from *M. bivittata*, meaning that these specimens could be a new species. More specimens should be studied to confirm this.

In Argentina, *M. bivittata* (Campbell et al., 2003) and *M. sanmartini* were recorded for *Marmosa cinerea* and *M. argentinensis* was recorded for *Thylamys pallidior* (see Jiménez et al., 2008). In Brazil, *M. bivittata* was reported parasitizing *D. albiventris* (see Justo et al., 2017). Other species of the genus *Didelphis* have been mentioned as hosts for *Mathevotaenia* sp. in Mexico and French Guiana (Monet-Mendoza et al., 2005; Jiménez et al., 2011). This is the first report of *Mathevotaenia* sp. from *D. aurita*.

#### Phylum Nemata Rudolphi, 1808

##### Order Trichinellida Hall, 1916

*Family Trichuridae Ramson, 1911*

*Trichuris minuta* (Rudolphi, 1819) (Babero, 1960) (Fig. 2C1, C2)

Description (6 males and 4 females): Mouth without lips; stichosome with 1 row of stichocytes. Bacillary band present. Spicule 0.82–1.14 long, spicule sheath 0.11–0.19 long, with spines less than 1 μm long, spicule tube 0.53–1.14 long, ejaculatory duct 0.95–1.65 long and testes 3.60–5.95 long. Vulva located at the 9.70–14.41 from the anterior end, vagina 0.90–1.60 long, ovary located at the 1.02–1.20 from the posterior end, uterus monodelphic, terminal anus. Eggs 0.06–0.07 long by 0.03–0.04 wide.

Taxonomy summary

*Host: Didelphis albiventris* (BH05, BH06, BH10, BH35, M174/22, M177/22, M211/23), *Didelphis aurita* (BH07, BH08, BH09, BH19, BH20, BH24, BH32).

*Infection site:* Large intestine and caecum.

*Localities:* Puerto Esperanza, Colonia Wanda, and Puerto Iguazú; Misiones, Argentina.

*Deposited material:* MLP-He 8143 (5 males and 5 females).

*Remarks*: Our specimens are morphologically like those described by Babero (1960). *Trichuris minuta* was originally described by Rudolphi (1819) parasitizing *Caluromys phylander* [originally mentioned as *Didelphis cayopollin* (Schreber, 1777)] in Brasilia, Brazil (Rudolphi, 1819). Later, Babero re-described the species from *D. virginiana* from Georgia, USA. More recently, this species was recorded in Brazil as a parasite of *D. albiventris* (Antunes, 2005) and *D. aurita* (see Noronha et al., 2002; Costa-Neto et al., 2018). This is the first report of *T. minuta* for Argentina.

*Trichuris didelphis* (Babero, 1960) (Fig. 2E)

*Description* (4 males): Anterior end very thin, mouth without lips. Stichosoma occupies approximately two-thirds of the length of the body; the body widens at the level of esophago-intestine junction. Spicule 0.89–0.20 long. Spicule sheath extending 0.20–0.30 beyond rear end of body, globular, pear-shaped in outline, with small spines less than 1 µm long. Cloaca 0.25–0.43 long, thick and muscular. Spicule tube 0.15–0.20 long. Ejaculatory duct 1.50–1.80 long and vas deferens 2.40–3.8 long.

Taxonomic summary

*Host: Didelphis aurita* (BH26, BH32, M220/23).

*Infection site:* Caecum.

*Locality*: Puerto Iguazú, Misiones, Argentina.

*Deposited material:* Specimens under study.

*Remarks*: This species was originally described by Babero (1960) from *D. virginiana* in Georgia, USA. In Brazil, it was also recorded in *D. albiventris* (see Silva and Costa, 1999; Antunes, 2005) and *D. aurita* (see Costa-Neto et al., 2018; Gentile et al., 2022). While in Argentina, it was recorded in *D. albiventris* (see Santa Cruz, 2006). Lombardero and Moriena (1973) reported the presence of *Trichuris* sp. in *D. albiventris* from Corrientes province. The drawings provided by these authors closely resemble *T. didelphis*, indicating that it might be the same species. This species was also reported in Mexico (Acosta-Virgen et al., 2015). This is the first report of *T. didelphis* parasitizing *D. aurita* in Misiones.

Family Capillaridae Raillet, 1915

*Capillaria* sp. 1 (Fig. 2F1, F2)

*Description* (5 females and 2 males): Small, slender-bodied nematodes, 11.2–16.50 total length; esophagus 5–5.3 long by 0.03–0.06 wide with well differentiated sticocytes and visible bacillary band; spicule 0.97–1 long and 0.01 wide, surrounded by a 0.67–0.9 spicule tube, covered by small cuticular spines; vulva located 4.45 from anterior region; eggs elongated barrel-shaped, colourless shell and operculum slightly projected outward, 0.05 by 0.03.

Taxonomic summary

*Host: Didelphis aurita* (BH08, BH32)

*Infection site:* Trachea, esophagus, stomach and small intestine.

*Locality:* Puerto Iguazú, Misiones, Argentina.

*Deposited material:* Specimens under study (retained by authors).

*Remarks*: Based on the morphological characters and the site of infection, we identified the specimen belonging to the genus *Capillaria*. In Brazil, several authors reported the presence of *Capillaria* sp. in the digestive tract of *D. albiventris* and other species of Didelphidae (Vicente et al., 1997; Silva and Costa, 1999; Noronha et al., 2002). Once again, these authors did not include good descriptions of the specimens. The genus *Capillaria* has a great diversity of species and many of them have been recorded from wild mammals (Butterworth and Beverley-Burton, 1977). Species identification of *Capillaria* is challenging due to the scarcity of males, the indistinct morphological traits of females and, in many cases, the limited availability of descriptions (López-Neyra, 1946). Moreover, Moravec (1982) argues that many of the descriptions of the genus *Capillaria* are erroneous. Herein, our specimens clearly correspond to *Capillaria* sp. because they were found in the digestive tract (Moravec, 1982). In Didelphidae previous studies have reported the presence of *C. longicauda* Freitas and Lent, 1935 in *P. opossum* and *D. marsupialis* (see López-Neyra, 1946; Jiménez et al., 2011). The spicule of *C. longicauda* has been described as poorly chitinized, transparent, and difficult to observe (López-Neyra, 1946). However, in this study the spicule was highly visible and well-chitinized, similarly to *Capillaria eberthi*. Further material and a revision of the specimens should be necessary to elucidate the identity of the species.

*Capillaria* sp. 2 (Fig. 2G)

*Description* (4 females): Nematodes small and slender; total length 7.75–10.05; oesophagus 3.15–4.25 long and 0.02–0.03 wide, with well-differentiated sticocytes, bacillary band absent; vulva at 3.85–4.3 from anterior end; eggs rounded, barrel-shaped, colourless shell with slightly outward projecting opercula, 0.06–0.07 by 0.03–0.04.

Taxonomy summary

*Host: Didelphis albiventris* (M177/22; M190/22)

*Infection site:* Stomach and small intestine.

*Localities*: Puerto Iguazú, Misiones, Argentina.

*Deposited material:* Specimens under study (retained by authors).

*Remarks*: Based on the morphological characters and the site of infection, we identified the specimen belonging to the genus *Capillaria*. In this case, only female specimens were recovered. *Capillaria* sp. 2 differ from *Capillaria* sp. 1 in body size, as they are considerably smaller; by the absence of the bacillary band, which was clearly observed in *Capillaria* sp. 1, and by the shape and size of the eggs, which are more rounded and larger than those of *Capillaria* sp. 1.

##### Order Strongylida Raillet y Henry, 1913

*Family Ancylostomatidae Looss, 1905*

*Globocephalus marsupialis* (Freitas de Teixeira and Lent, 1936) (Fig. 2H1, H2)

*Description* (10 males and 10 females): White body with transversely striated cuticle. Subglobular stoma, with a pair of large sub-ventral teeth near the base of the capsule. Well-developed claviform muscular oesophagus. Spicules 0.32–0.45 long and gubernaculum 0.03–0.07 long. Female with amphidelphic uterus. Vulva at 4–5.25 from the anterior end. Eggs 0.084–0.11 long by 0.05–0.06 wide.

Taxonomy summary

*Host: Didelphis albiventris* (M190/22)

*Infection site:* Small intestine.

*Locality:* Puerto Iguazú, Misiones, Argentina.

*Deposited material:* MLP-He 8145 (5 males and 5 females).

*Remarks:* The specimens herein studied are consistent with the morphological characteristics given by Freitas de Teixeira and Lent (1936) parasitizing *Metachirus myosurus* Temminck, 1824 from Brazil. It was also recorded in *D. aurita* (see Costa-Neto et al., 2018; Gentile et al., 2022). This is the first report of *G. marsupialis* parasitizing *D. albiventris* in Argentina.

Family Viannaiidae Neveu-Lemaire, 1944

*Viannaia viannai* (Travassos, 1914) (Fig. 2I)

*Description* (8 males and 6 females): nematodes small and thin, body curled around its own axis. Synlophe without lateral or dorsal ridges. Cephalic extremity with a highly developed cuticular expansion. Males with highly developed caudal bursa, with 2 slightly asymmetrical lateral lobes and highly developed dorsal lobe. Bursa 2–1–2 type. Spicules 0.12–0.158 long. Females monodelphic. Vulva at 1.25–2.8 from posterior end. Eggs 0.05–0.07 long by 0.04–0.05 wide.

Taxonomic summary

*Host: Didelphis albiventris* (BH35; M190/22, M211/23), *Didelphis aurita* (BH07, BH08, BH09, BH21, BH24, BH32, BH34, M220/23).

*Infection site:* Small intestine.

*Localities*: Puerto Iguazú, Misiones, Argentina.

*Deposited material:* MLP-He 8144 (5 males and 5 females).

*Remarks*: The specimens fit the description provided by Travassos (1914). In Brazil, *V. viannai* was reported in *D. aurita* (see Travassos, 1914) and *P. opossum* (see Corrêa Gomes et al., 2003); and in Bolivia it was mentioned parasitizing 6 species of Didelphidae (see Jiménez et al., 2024). It was also reported from Peru, Mexico, Venezuela, and French Guyana (Guerrero, 1985; Monet-Mendoza et al., 2005; Jiménez et al., 2011). This is the first report of *V. viannai* parasitizing *Didelphis* spp. in Argentina.

*Travassostrongylus orloffi* Travassos, 1935 (Fig. 2J1, J2).

*Description* (4 males and 5 females): Small nematodes. Cuticle with transverse striations and longitudinal lines; synlophe with 10 symmetric crests oriented from right to left. Cephalic cuticular dilatation of 0.09–0.13. Bursa 2–2–1 type. Spicules subequal, complex, 0.12–0.14 long by 0.02–0.04 wide, distal end bifid; gubernaculum 0.06–0.11 long; conical telamon 0.03–0.07 long. Vulva 1.05–1.2 from posterior end, anus 0.1–0.14 from posterior end. Eggs 0.05–0.06 long by 0.04–0.05 wide.

Taxonomic summary

*Host: Didelphis aurita* (BH32)

*Infection site:* Small intestine

*Localities*: Puerto Iguazú, Misiones, Argentina.

*Deposited material:* Specimens under study (retained by authors).

*Remarks*: Our specimens are morphologically similar to those described by Diaw (1976). Descriptions for this species by Travassos (1937) and Diaw (1976) mention the presence of a subconical telamon. In this work, we also note that the telamon exhibits bilateral projections at one-third of its height. In Brazil, *T. orloffi* was reported for *D. albiventris* (see Silva and Costa, 1999; Antunes, 2005) and *D. aurita* (see Costa-Neto et al., 2018; Gentile et al., 2022). It was also found in French Guyana (Diaw, 1976) and Mexico (Scheibel et al., 2014). This is the first record of *Travassostrongylus orloffi* for *D. aurita* in Argentina.

*Travassostrongylus callis* (Travassos, 1914) Orloff, 1933 (Fig. 2L1, L2)

*Description*: (7 males and 5 females): Nematodes small, cuticle with transverse and longitudinal striations, cephalic cuticular dilatation 0.1–0.11. Bursa 2–2–1 type. Spicules subequal, complex, 0.1–0.5 long and 0.01–0.02 wide, gubernaculum 0.04–0.05 long, conical telamon 0.03–0.05 long, with a left hook. Vulva located 1–1.4 from hind end, with a cuticular cap. Anus 0.1–0.13 from posterior end. Eggs 0.05 × 0.03.

Taxonomic summary

*Host: Didelphis albiventris* (M211/23), *Didelphis aurita* (BH32, M220/23)

*Infection site*: Small intestine

*Localities*: Puerto Iguazú, Misiones, Argentina.

*Deposited material*: Specimens under study (retained by authors).

*Remarks:* Our specimens are similar with those described by Travassos (1937) and Diaw (1976). *Travassostrongylus callis* was originally found in *D. aurita* from Brazil (Travassos, 1914). Later, it was mentioned from other localities from Brazil (Noronha et al., 2002), and recently Jiménez et al. (2024) mentioned it for *Chironectes minimus* Zimmermann, 1780 in Bolivia. It was also found parasitizing *D. marsupialis* in French Guiana (Diaw, 1976), and Panama (Scheibel et al., 2014). This is the first report in *D. albiventris* and *D. aurita* from Argentina.

##### Order Ascaridida Chabaud, 1965

*Superfamily Heterakoidea Railliet and Henry, 1912*

Family Aspidoderidae Skrajabin y Schikhobalova, 1947

*Aspidodera raillieti* Travassos, 1913 (Fig. 2K1, K2)

*Description* (9 males and 9 females): Anterior end with a cap with 6 longitudinal loops or cords, 3 of them on the interlips and 3 on each lip. Oesophagus with a terminal bulb. Spicules similar in shape and size, 0.55–0.93 long. Gubernaculum 0.15–0.22 long. Cloaca at 0.28–0.38 from posterior end. Vulva at 1.90–2.65 from posterior end, amphidelphic uterus. Eggs 0.05–0.07 long by 0.04–0.05 wide.

Taxonomic summary

*Host: Didelphis albiventris* (BH35, M174/22), *Didelphis aurita* (BH07, BH08, BH09, BH20, BH21, BH32, M220/23).

*Infection site*: Large intestine and caecum

*Localities*: Puerto Iguazú, Misiones, Argentina.

*Deposited material*: MLP-He 8147 (5 males and 5 females).

*Remarks*: Nematodes similar in morphology to those described by Portes Santos et al. (1990) and (Chagas-Moutinho et al., 2007). The specimens characterized by Chagas-Moutinho et al. (2007) are relatively smaller and we consider that the differences are induced by the host and geographic distance. *Aspidodera raillieti* was also recorded in several species of marsupials from Brazil (Costa-Neto et al., 2018; Gentile et al., 2022), Bolivia, Paraguay and Mexico (Jiménez et al., 2024), French Guiana (Jiménez et al., 2011), Peru (Polo-González et al., 2019), and other localities. In Argentina, *A. raillieti* was only recorded in *D. albiventris* (see Lombardero and Moriena, 1973; Navone and Suriano, 1992). This is the first record for *D. aurita* in Argentina.

Family Kathlaniidae Lane, 1914

*Cruzia tentaculata* (Rudolphi, 1819) (Travassos, 1922) (Fig. 2M)

*Description* (20 males and 20 females): Oral opening surrounded by 3 lips, 1 dorsal and 2 latero-ventral; inner margin of lips with small, serrated structures. Oesophagus ending in a bulb with an intestinal caecum projecting anteriad beyond the bulb. Spicules 0.80–1.20 long. Gubernaculum 0.18–0.40. Cloaca at 0.10–0.20 from posterior end. Vulva located at the middle of the body 4.35–9.40 from the anterior end. Anus at 0.55–1.90 from the posterior end. Eggs 0.10–0.15 long by 0.5–0.07 wide.

Taxonomic summary

*Host: Didelphis albiventris* (BH01, BH05, BH06, BH10, M128/22, BH35, M82/21, M129/22, M174/22, M177/22, M190/22, M211/23), *Didelphis aurita* (BH07, BH08, BH09, BH19, BH20, BH21, BH24, BH26, BH32, BH34, M220/23).

*Infection site*: Large intestine and caecum.

*Localities*: Puerto Esperanza, Colonia Wanda, Puerto Iguazú, Misiones, Argentina.

*Deposited material:* MLP-He 8146 (5 males and 5 females).

*Remarks: Cruzia tentaculata* was originally described parasitizing *D. aurita* from Brazil (Travassos, 1922). After that, it was recorded in several South American didelphids (see Noronha et al., 2002; Corrêa Gomes et al., 2003; Adnet et al., 2009; Costa-Neto et al., 2018; Jiménez et al., 2024). In Argentina, it is mentioned parasitizing *D. albiventris* and *L. crassicaudata* (see Boero and Boehringer, 1967; Santa Cruz, 2006). This is the first record for *D. aurita* in Argentina.

##### Order Spirurida Railliet, 1915

*Family Physalopteridae Railliet, 1893*

*Turgida turgida* (Rudolphi 1819) Travassos, 1919

*Description* (5 males and 5 females): Large nematodes with a rigid whitish body, covered by a thick cuticle with fine transverse striations. Oral opening surrounded laterally by 2 symmetrical, semi-domed lips or pseudolabia, composed of 3 fused lips that are flattened on the inner part. A cephalic collarette, formed by a deep fold surrounds the pseudolabia. Male tail with caudal alae with 22 papillae: 4 pairs of pedunculated papillae are placed on the caudal alae; 3 papillae are located anteriorly to the cloaca; spicules 0.35–0.60 long; tail 0.3–0.9 length. Vulva is located below the end of the oesophagus at 4.84–9.10 from the anterior end; tail 0.3–0.7 length.

Taxonomic summary

*Host: Didelphis albiventris* (BH01, M129/22, M190/22, M211/23), *Didelphis aurita* (BH07, BH19, BH20, BH21, BH26).

*Infection site*: Stomach

*Localities:* Colonia Wanda, and Puerto Iguazú, Misiones, Argentina.

*Deposited material*: MLP-He 8148 (5 males and 5 females)

*Remarks: Turgida turgida* was recorded parasitizing *Didelphis* spp. from North and South America (Humberg et al., 2011). Our specimens are morphologically like those described by Travassos (1920), Matey et al. (2001), and Humberg et al. (2011). In Brazil, *T. turgida* was recorded in *D. albiventris, D. aurita, C. minimus* and *P. quica* Temminck, 1824 (see Noronha et al., 2002; Humberg et al., 2011; Costa-Neto et al., 2018); from Bolivia in *D. albiventris* and *P. opossum* (see Jiménez et al., 2024), while in Argentina it was reported parasitizing *D. albiventris* and *L. crassicaudata* (see Boero and Boehringer, 1967; Navone and Suriano, 1992; Santa Cruz, 2006). This is the first record for *D. aurita* in Argentina.

#### Phylum Acanthocephala Kohlreuther, 1771

##### Clase Archiacanthocephala Meyer, 1931

*Orden Oligacanthorhynchida Petrochenko, 1956*

Familia Oligacanthorhynchidae Southwell et Macfie, 1925

*Oligacanthorhynchus microcephalus* (Rudolphi, 1819) Schmidt, 1972 (Fig. 2N)

*Description* (5 females): Individuals of large size, cuticle striated with transverse roughness. Proboscis small, ovoid with 5–6 semiuniform double-rooted hooks of 75–100 µm length. Distinct neck, its length and width depend on the degree of extension of the trunk and proboscis. Small, elliptical embryonated eggs surrounded by a thick outer membrane of 80–100 µm long by 40–50 µm wide.

Taxonomic summary

*Host: Didelphis albiventris* (BH10, M128/22, BH35, M129/22, M174/22, M177/22, M190/22, M211/23) and *Didelphis aurita* (BH20, BH21, BH24, M220/23).

*Infection site*: Small intestine.

*Localities*: Puerto Esperanza, Puerto Iguazú, Misiones, Argentina.

*Deposited material*: MLP-He 8149 (5 males and 5 females).

*Remarks*: The identification was based on Richardson et al. (2014). *Oligacanthorhynchus microcephalus* is distributed in North and South America and it parasitizes members of Didelphimorphia as its unique definitive hosts (Richardson et al., 2014). In the serosa, white nodules were evident indicating the characteristic lesions affecting the mucosa, submucosa, and muscle layers of the marsupial’s intestine (Richardson and Barnawell, 1995). In Argentina, this species has been mentioned as a parasite of *D. albiventris* (see Boero and Boehringer, 1967; Navone and Suriano, 1992). In Brazil it was cited for *D. albiventris, D. aurita* and *M. myosurus* (see Carneiro de Souza et al., 2017; Zabott et al., 2017; Costa-Neto et al., 2018; Cirino et al., 2020) and from Bolivia in *M. opossum* and *P. opossum* (see Jiménez et al., 2024).

#### Ecological descriptors

##### Characterization of the infections

During our collections we found a total of 15 species of helminths. The specific richness in *D. albiventris* was 12, while in *D. aurita* was 13 (see Table 1). Both species of *Didelphis* share 10 species of helminths; *D. albiventris* presented *Capillaria* sp. 2 and *G. marsupials*, absent in *D. aurita*; while *D. aurita* presented *T. didelphis, Capillaria* sp. 1 and *T. orloffi*, absent in *D. albiventris*.

In *D. albiventris, C. tentaculata* registered the highest prevalence (P), followed by *O. microcephalus* and *T. minuta*; the lower P were registered for *G. marsupialis* and *Mathevotaenia* sp. (see Table 1). Related to the MI and MA, *C. tentaculata, Mathevotaenia* sp. and *O. microcephalus* showed the highest values. In *D. aurita, C. tentaculata* registered the highest P, followed by *V. viannai, T. minuta, A. raillieti* and *B. migrans*; while the lower P were registered for *Mathevotaenia* sp. and *T. orlofi*. The highest values of MI and MA correspond to *C. tentaculata, V. viannai*, and *R. coronatus*.

Most of the helminths recovered display direct life cycles, except for *R. coronatus, B. migrans, Mathevotaenia* sp., *C. tentaculata, T. turgida* and *O. microcephalus* (see Table 1).

## Discussion

In this study we examined the parasite community of 2 sympatric species of opossums in northern Misiones. We present new records for Argentina including *T. minuta, G. marsupialis, V. viannai, T. orlofi*, and *T. callis*. In *D. aurita*, we identified 3 species of Platyhelminthes, 9 nematodes, and 1 acanthocephalan, all of which are new records for the country.

In *D. albiventri*s, 6 out of the 12 helminth species identified have an indirect life cycle. Similarly, in *D. aurita*, 6 out of 13 helminth species exhibit an indirect life cycle (see Table 1). This suggests that nearly half of the helminth species in both opossum species need an intermediate host acquired through the diet. Metacercariae of *Brachylaima* sp. have been reported in the land slug *Phyllocaulis variegatus* from Misiones (Valente et al., 2016), while those of *Rhopalias* sp. were found encysted in tadpoles of *Rhinella* sp. from the same stream where planorbid snails harboured cercariae (López-Hernández et al., 2023), suggesting that slugs and amphibians respectively, serve as intermediate hosts from these trematodes. The genus *Mathevotaenia* includes several species that have been found parasitizing mammals worldwide (i.e. rodents, insectivores, edentates, marsupials, bats), with isolated reports in reptiles and birds (Beveridge, 2008; Bursey et al., 2010; Lunaschi et al., 2012). Spasskii (1951) suggested that the life cycle of *Mathevotaenia* species involve insects, such as cockroaches and butterflies as intermediate hosts. Moreover, the nematode *T. turgida* uses insects of the orders Orthoptera and Coleoptera as intermediate hosts and is specific for certain species of opossums (Anderson, 2000). Furthermore, native snails of the genus *Thaumastus* and *Latipes*, plus the invasive snail *Achatina fulica* in Brazil, were found to be the intermediate host of *C. tentaculata* (Ramos de Souza et al., 2021). Richardson et al. (2014) identified millipedes of the genus *Narceus* as natural intermediate hosts for the acanthocephalan *O. microcephalus*. This raises the possibility that millipedes in the study area could also serve as intermediate hosts for this species.

Although there are few studies on the feeding habits of opossums, existing research suggests they are primarily insectivorous (Kasparian et al., 2002; Richardson, 2006). Sandidge (1953) analysed the stomach contents of *D. virginiana* and found not only insects but also mammals, birds, reptiles, amphibians, and, to a lesser extent, centipedes, snails, and crayfish. Given this diversity in diet and the high prevalence of helminths with indirect life cycles found in the present study, plus the finding of legs and antenna of insects, birds and mouse hair in the digestive tracts of the studied host, we suggest that *D. aurita* and *D. albiventris* may consume slugs, snails, millipedes, and other vertebrates as part of their diet, indicating that these prey items act as intermediate hosts of the parasite species reported herein, constituting a significant portion of their prey in the study area.

The 2 host species studied herein share 10 helminth taxa. *Didelphis aurita* occurs both, in Rio de Janeiro and in Misiones, and in both localities share a substantial number of species (8 over 14 parasite taxa) (Costa-Neto et al., 2018; Gentile et al., 2022). This pattern aligns with findings from similar studies on sympatric opossum species in French Guiana and Mexico (Jiménez et al., 2011). Research shows that parasite communities in the same locality often exhibit higher taxonomic similarity than those in conspecific species of marsupials living in different areas. This can be attributed to the generalist feeding habits and overlapping diets of sympatric opossums, which expose them to the same parasites or to ecological fitting where the parasites shift to a new host and adapt or survive to the new association (Combes, 1991; Agosta et al., 2010). In the study area, *D. albiventris* and *D. aurita* have been observed living in sympatry (Cruz et al., 2019). Moreover, closely related species often share physiological, immunological, and ecological traits, making them compatible hosts for the same parasites (Combes, 1991; Krasnov et al., 2006; Poulin, 2014). Therefore, the sympatry, shared diet, and the conspecificity of these opossums likely explain the similarities observed in their helminth communities.

Knowledge of wildlife parasitology is essential for understanding the complex relationships between parasites and their animal hosts in natural ecosystems. Our research aims to provide crucial data on 2 host species poorly studied and frequently observed in the environment as well as in road-killed routes. Considering the habitat fragmentation in the region, the locally disturbances in the study area, and that both host species have been reported as reservoirs of zoonotic pathogens in other regions (Castaño Zubieta et al., 2014; Bezerra-Santos et al., 2021), this study provides important information in the southern distribution of both opossum’s species for the AF. None of the helminths identified herein are zoonotic for humans or domestic animals. Complementary molecular studies from the different helminth species will be crucial in elucidating the identity of unclear taxa such as *Capillaria* or *Mathevotaenia*.

## Appendix

Data on locality and host species studied. Acronyms: BH: Barbara Hartmann field number; M: Dante Di Nucci catalogue. CM-LGE: Colección de Mastozoología del Laboratorio de Genética Evolutiva – Dr Claudio Bidau from Instituto de Biología Subtropical. MLP-He: Colección de Helmintología from Museo de La Plata.

**Table:**

	Locality	Zone	Lat (S)	Long (W)	Host collection no.	Host field no.	Sex	Helminths collection no.
*Didelphis albiventris*	Puerto Iguazú	Villa Florida Neighbourhood	25º35´55´´	54º34´38´´	M82/21		Male	
					M128/22		Female	
					M190/22		Female	MLP-He 8145MLP-He 8145
					M211/23		Female	MLP-He 8149MLP-He 8149
		300 Viviendas Neighbourhood	25º36´50´´	54º34´02´´	M129/22		Female	
		Villa 14 Neighbourhood	25º35´53´´	54º34´08´´	M177/22		Male	
		Zona de Granjas Neighbourhood	25º37´02´´	54º34´01´´	BH35		Female	
	Colonia Wanda	NR 12, Km 1592	25º58´44´´	54º34´49´´	BH01		Female	
		PR 19, Km 13	25º58´34´´	54º30´49´´	BH06		Female	
	Puerto Esperanza	Paraje Puerto Helvecia	26º03´04´´	54º38´20´´	BH05		Female	
		Alegre Neighbourhood	26º01´42´´	54º35´12´´	BH10		Female	
*Didelphis aurita*	Puerto Iguazú	San Martin Avenue	25º35´44´´	54º34´21´´	BH26		Female	
	Iguazú National Park	NR 12	25º39´60´´	54º30´06´´	BH07		Female	
		NR12, Km 1931	25°39´54´´	54º30´14´´	BH09		Male	
		NR 12	25°40´22´´	54°30´07´´	BH19		Female	
		NR 12, Km 1633	25°38´19´´	54º32´11´´	BH21		Female	MLP-He 8148 MLP-He 8148
		NR 101	25°38´19´´	54°28´42´´	BH08		Male	
			25°40´46´´	54°29´17´´	BH24		Male	
			25°40´17´´	54°29´44´´	BH32		Male	MLP-He 8144 MLP-He 8146 MLP-He 8146 MLP-He 8147 MLP-He 8147 MLP-He 8144
			25°40´26´´	54°29´32´´	BH34		Male	
			25°41´08´´	54°28´58´´	M220/23		Male	
			25°41´28´´	54°28´41´´	BH20	CM-LGE 898	Female	MLP-He 8141 MLP-He 8142 MLP-He 8143 MLP-He 8143

## References

[ref1] Acosta-Virgen K, López-caballero J, García-Prieto L and Mata-López R (2015) Helminths of three species of opossums (Mammalia, Didelphidae) from Mexico. *ZooLeys* 511, 131–152. doi:10.3897/zookeys.511.9571 PMC4523750 26257556

[ref2] Adnet FAO, Anjos DHS, Menezes-Oliveira A and Lanfredi RM (2009) Further description of *Cruzia tentaculata* (Rudolphi, 1819) Travassos, 1917 (Nematoda: Cruzidae) by light and scanning electron microscopy. *Parasitology Research* 104, 1207–1211. doi:10.1007/s00436-008-1316-6 19130086

[ref3] Agosta SJ, Janz N and Brooks DR (2010) How specialists can be generalists: resolving the “Parasite Paradox” and implications for emerging infectious disease. *Zoologia* 27, 151–162. doi:10.1590/S1984-46702010000200001

[ref4] Anderson RC (2000) *Nematode Parasites of Vertebrates. Their Development and Transmission*. 2nd edn. Wallingford: CABI Publishing, 672.

[ref5] Antunes GM (2005) Diversidade e potencial zoonotico de parasitos de *Didelphis albiventris*. PhD thesis, Universidade Federal do Rio Grande Do Sul, Brazil.

[ref6] Babero BB (1960) Further studies on helminths of the opossum, *Didelphis virginiana*, with a description of a new species from this host. *Journal of Parasitology* 46, 455–463. 13795484

[ref7] Beveridge I (2008) *Mathevotaenia niuguiniensis* n. sp. (Cestoda: Anoplocephalidae: Linstowiinae) from the water-rat *Parahydromys asper* (Thomas) in Papua New Guinea, with a list of species of *Mathevotaenia* Akumyan, 1946. *Systematic Parasitology* 71, 189–198. doi:10.1007/s11230-008-9155-5 18815898

[ref8] Bezerra-Santos MA, Ramos RAN, Campos AK, Dantas-Torres F and Otranto D (2021) *Didelphis* spp. opossums and their parasites in the Americas: a one health perspective. *Parasitology Research* 120, 4091–4111. doi:10.1007/s00436-021-07072-4 33788021 PMC8599228

[ref9] Boero JJ and Boehringer IK (1967) El parasitismo de nuestra fauna autóctona. Los Parásitos de la Comadreja Picaza (*Didelphis azarae*) y de la Comadreja Colorada (*Lutrolina crassicaudata*). *Revista de La Facultad de Ciencias Veterinarias de La Plata* 21, 147–160.

[ref10] Bursey CR, Goldberg SR and Telford SR (2010) A new species of *Mathevotaenia* (Cestoda, Anoplocephalidae, Linstowiinae) from the lizard *Sceloporus malachiticus* (Squamata, Phrynosomatidae) from Panama. *Acta Parasitologica* 55, 53–57. doi:10.2478/s11686-010-0001-y

[ref11] Bush OA, Lafferty KD, Lotz JM and Shostak AW (1997) Parasitology on its own terms: Meets ecology Margolis. *The Journal of Parasitology* 83, 575–583. 9267395

[ref12] Butterworth EW and Beverley-Burton M (1977) *Capillaria didelphis n. sp.* (Nematoda: Trichuroides) from the Opossum, *Didelphis virginiana* L. in Georgia. *Canadian Journal of Zoology* 30, 616–619.

[ref13] Campbell ML, Gardner SL and Navone GT (2003) A new species of *Mathevotaenia* (Cestoda: Anoplocephalidae) and other tapeworms from Marsupials in Argentina. *Journal of Parasitology* 89, 1181–1185. 14740908 10.1645/GE-1778

[ref14] Carneiro de Souza A, Furtado AE, Stutz RS, Santos NA, Martins BGT, da Silva ME, et al (2017) First report of *Oligacanthorhynchus microcephalus* (Rudolphi, 1819) (Acanthocephala: Oligacanthorhynchidae) in *Didelphis albiventris* (Lund, 1841) (Marsupialia: Didelphidae) in Southeastern Brazil. *Journal of Dairy, Veterinary & Animal Research* 5, 3–6. doi:10.15406/jdvar.2017.05.00143

[ref15] Castaño Zubieta R, Ruiz M, Morici G, Lovera R, Fernández MS, Caracostantogolo J and Cavia R (2014) First report of *Trichinella spiralis* from the White-Eared (*Didelphis albiventris*) and the thick-tailed opossum (*Lutreolina crassicaudata*) in Central Argentina. *Helminthologia* 51, 198–202. doi:10.2478/s11687-014-0229-4

[ref16] Chagas-Moutinho VA, Oliveira-Menezes A, Cárdenas MQ and Lanfredi MR (2007) Further description of *Aspidodera raillieti* (Nematoda: Aspidoderidae) from *Didelphis marsupialis* (Mammalia: Didelphidae) by light and scanning electron microscopy. *Parasitology Research* 101, 1331–1336. doi:10.1007/s00436-007-0641-5 17622560

[ref17] Chemisquy MA and Martin GM (2019) *Didelphis albiventris*. In SAyDS–SAREM (ed.), *Categorización 2019 de los mamíferos de Argentina según su riesgo de extinción. Lista Roja de Los Mamíferos de Argentina*. Argentina: Sarem 1–9. https://cma.sarem.org.ar/es/especie-nativa/didelphis-albiventris

[ref18] Chero JD, Sáez G, Mendoza-Vidaurre C, Iannacone J and Cruces CL (2017) Helminths of the common opossum *Didelphis marsupialis* (Didelphimorphia: Didelphidae), with a checklist of helminths parasitizing marsupials from Peru. *Revista Mexicana de Biodiversidad* 30, 1–12. doi:10.1016/j.rmb.2017.07.004

[ref19] Cirino BS, Costa Neto SF, Maldonado A, Jr. and Gentile R (2020) First study on the helminth community structure of the neotropical marsupial *Metachirus myosuros* (Didelphimorphia, Didelphidae). *Revista Brasileira de Parasitologia Veterinaria* 29, 1–13. doi:10.1590/s1984-29612020064 32876091

[ref20] Combes C (1991) Evolution of parasite life cycles. In Toft CA, Aeschlimann A and Bolis L (eds.), *Parasite-host Associations. Coexistence or Conflict?* Oxford: Oxford University Press, 319.

[ref21] Corrêa Gomes D, da Cruz R P, Vicente JJ and Magalhães Pinto R (2003) Nematode parasites of marsupials and small rodents from the Brazilian Atlantic Forest in the State of Rio de Janeiro, Brazil. *Revista Brasileira de Zoologia* 20, 699–707. doi:10.1590/s0101-81752003000400024

[ref22] Costa-Neto SF, Cardoso TS, Boullosa RG, Maldonado A and Gentile R (2018) Metacommunity structure of the helminths of the Black-Eared Opossum *Didelphis aurita* in peri-urban, sylvatic and rural environments in south-eastern Brazil. *Journal of Helminthology* 93, 720–731. doi:10.1017/S0022149X18000780 30220264

[ref23] Cruz P, Iezzi ME, De Angelo C, Varela D and Di Bitetti MS (2019) Landscape use by two opossums is shaped by habitat preferences rather than by competitive interactions. *Journal of Mammalogy* 100, 1966–1978. doi:10.1093/jmammal/gyz133

[ref24] Diaw OT (1976) Contribution à l’étude de nématodes Trichostrongyloidea parasites de Xenarthre, Marsupiaux et Rongeurs Néotropicaux. *Bulletin Du Muséum National D’histoire Naturelle* 405, 1065–1089. doi:10.5962/p.281416

[ref25] Freitas de Teixeira JF and Lent H (1936) Estudo sobre o generO Globo*cephalus* Molin, 1861 (Nematoda: Strongyloidea). *Memorias Do Instituto Oswaldo Cruz* 31, 69–83.

[ref26] Gentile R, Costa-Neto SF, Cardoso T and Maldonado A (2022) Helminths of small mammals in an Atlantic forest biological station in Rio de Janeiro, Brazil. *Neotropical Helminthology* 16, 161–172. doi:10.24039/rnh20221621451

[ref27] Guerrero R (1985) Nematoda: Trichostorngyloidea parásitos de mamíferos silvestres de Venezuela. II Revisión del género *Viannaia* Travassos, 1914. *Memoria de La Sociedad de Ciencias Naturales La Salle* 45, 9–47.

[ref28] Hartmann BB (2023) Helmintos de *Didelphis albiventris* y *Didelphis aurita* (Mammalia, Didelphimorphia) en ambientes selváticos y antrópicos en el Departamento Iguazú, Misiones. *Revista de Mastozoología Neotropical* 30, 96.

[ref29] Haverkost TR and Gardner SL (2008) A review of species in the genus *Rhopalias* (Rudolphi, 1819). *Journal of Parasitology* 94, 716–726. 18605801 10.1645/GE-1423.1

[ref30] Humberg RMP, Tavares LER, Paiva F, Oshiro ET, Bonamigo RA, Júnior NT and Oliveira AG (2011) *Turgida turgida* (Nematoda: Physalopteridae) parasitic in White-Bellied opossum, *Didelphis albiventris* (Marsupialia: Didelphidae), state of Mato Grosso Do Sul, Brazil. *Pesquisa Veterinária Brasileira* 31, 78–80. doi:10.1590/s0100-736x2011000100012

[ref31] Jiménez AF, Campbell ML, Byles B, Scheibel PG and Gardner SL (2024) Gastrointestinal helminths of opossums (Mammalia: Didelphidae) from Bolivia. *Parasitology*, 1–53. doi:10.1017/S0031182024000490 PMC11474012 38682282

[ref32] Jiménez FA, Braun JK, Campbell ML and Gardner SL (2008) Endoparasites of fat-tailed mouse opossums (*Thylamys*: Didelphidae) from northwestern Argentina and southern Bolivia, with the description of a new species of tapeworm. *Journal of Parasitology* 94, 1098–1102. doi:10.1645/GE-1424.1 18973415

[ref33] Jiménez FA, Catzeflis F and Gardner SL (2011) Structure of parasite component communities of *Didelphis marsupials*: Insights from a comparative study. *Journal of Parasitology* 97, 779–787. doi:10.1645/GE-2711.1 21506798

[ref34] Justo MCN, Fernandes BMM, Knoff M, Cárdenas MQ and Cohen SC (2017) Checklist of brazilian Cestoda. *Neotropical Helminthology* 11, 187–282.

[ref35] Kasparian M, Hellgren EC and Ginger SM (2002) Food habits of the Virginia opossum during raccoon removal in the cross timbers ecoregion, Oklahoma. *Wildlife Research* 78, 73–78.

[ref36] Krasnov BR, Poulin R and Morand S (2006) Patterns of macroparasite diversity in small mammals. *Micromammals and Macroparasites: From Evolutionary Ecology to Management*, 197–231. doi:10.1007/978-4-431-36025-4_12

[ref37] Lombardero OJ and Moriena RA (1973) Nuevos helmintos de la comadreja overa (*Didelphis azarae*) para la Argentina. *Revista de Medicina Veterinaria* 53, 315–320.

[ref38] López-Hernández D, Caixeta Valadão M, Lane de Melo A, Tkach VV and Alves PH (2023) Elucidating the life cycle of opossum parasites: DNA sequences reveal the involvement of planorbid snails as intermediate hosts of *Rhopalias* spp. (Trematoda: Echinostomatidae) in Brazil. *PLoS One* 18, 1–18. doi:10.1371/journal.pone.0279268 PMC9983843 36867609

[ref39] López-Neyra (1946) Tomo XII: Los Capilariinae. In *Tomo XII: Los Capilariinae* Valverde, Madrid: Memorias de La Real Academia de Ciencias Exactas, Físicas Y Naturales, 262.

[ref40] Lunaschi LI and Drago FB (2007) Checklist of digenean parasites of wild mammals from Argentina. *Zootaxa* 50, 35–50. doi:10.11646/zootaxa.1580.1.3

[ref41] Lunaschi LI, Lamas MF and Drago FB (2012) A new species of *Mathevotaenia* (Cestoda, Anoplocephalidae) parasitizing *Tropidurus spinulosus* (Reptilia, Squamata) from northeastern Argentina. *Revista Mexicana de Biodiversidad* 83, 583–590. doi:10.7550/rmb.27660

[ref42] Marinho de Quadros R, Müller G, Barbosa de Moura A, Rodríguez RB, Martins KH, Filippi DA and Mazzoni B (2016) *Rhopalias coronatus* (Rudolphi, 1819) Stiles and Hassall 1898 in *Didelphis albiventris* from Santa Catarina, Brasil. *Parasitología Latinoamericana* 65, 38–41.

[ref43] Martínez AH, Brandetti E and Boero JJ (1973) El parasitismo de nuestra fauna autóctona. Hallazgo de dos nuevas especies de trematodes, *Rhopalias baculifer* Braun, 1900 y *Rhopalias coronatus* Stiles y Hassal, en comadrejas colorada (*Lutreolina crassicaudata*) y picaza (*Didelphis azarae*). *Revista de Medicina Veterinaria* 54, 69–73.

[ref44] Martínez FA (1986) Helmintofauna de los mamíferos terrestres. Trematodes. *Veterinaria Argentina* 3, 544–551.

[ref45] Massoia E, Chebez JC and Bosso A (2012) *Mamíferos silvestres de la provincia de Misiones, Argentina*. 1st edn. Buenos Aires: Fundación de Historia Natural Félix de Azara, 510. https://www.fundacionazara.org.ar/img/libros/los-mamiferos-silvestres-de-la-provincia-de-misiones.pdf

[ref46] Matey VE, Kuperman BI and Kinsella JM (2001) Scanning electron microscopy of *Turgida turgida* (Nematoda: Spiruroidea), parasite of the Virginia opossum, *Didelphis virginiana*, from southern California. *Journal of Parasitology* 87, 1199–1202. doi:10.2307/3285267 11695400

[ref47] Monet-Mendoza A, Osorio-Sarabia D and Garcia-Prieto L (2005) Helminths of the Virginia opossum *Didelphis virginiana* (Mammalia: Didelphidae) in Mexico. *Journal of Parasitology* 91, 213–219. doi:10.1645/GE-273R 15856909

[ref48] Montes de Oca M, Dominguez MG, Lammel MN and Cavia R (2024) Natural Trichinella spiralis infection in wild and domestic vertebrates in a trichinellosis endemic area from Argentina. *Mastozoología Neotropical* 31. doi:10.31687/saremMN.24.31.01.22.e0993

[ref49] Moravec F (1982) Proposal of a new systematic arrangement of Nematodes of the Family Capillariidae. *Folia Parasitologica* 29, 119–132. 7106653

[ref50] Myers N, Mittermeier RA, Mittermeier CG, da Fonseca GAB and Kent J (2000) Biodiversity hotspots for conservation priorities. *Nature* 403, 853–558. 10.1038/35002501 10706275

[ref51] Navone GT (1989) *Pterygodermatites* (Paucipectines) *kozeki* (Chabaud et Bain, 1981) n. comb., parásito de *Lestodelphys halli* Tate, 1934, *Didelphis albiventris* L. y *Thylamis pusilla* (Desmarest) de la República Argentina. Anatomía y posición sistemática. *Revista Ibérica de Parasitología* 49, 219–226.

[ref52] Navone GT and Suriano DM (1992) Species composition and seasonal dynamics of the helminth community parasitizing *Didelphis albiventris* (Marsupialia: Didelphidae) in savannas of the central Argentina. *Ecología Austral* 2, 95–100.

[ref53] Navone GT, Suriano DM and Pujol CA (1991) *Travassostrongylus yungaensis* n. sp. and *Hoineffia simplicispicula* n. sp. (Nematoda: Trichostrongyloidea) from *Thylamys venustus cinderellus* and *Lutreolina crassicaudata* (Marsupialia: Didelphidae) in the northwest of Argentina. *Systematic Parasitology* 19, 187–193. doi:10.1007/BF00011886

[ref54] Noronha D, Vicente JJ and Pinto EM (2002) A survey of new host records for nematodes from mammals deposited in the Helminthological Collection of the Oswaldo Cruz Institute (CHIOC). *Revista Brasileira de Zoologia* 19, 945–949.

[ref55] Pastrán-López OGA, Rivero-Castro AG, Ruiz-Estebez EG, Amoni-Sacchi HJ and Sánchez-Castro L (2022) Ampliación de distribución de *Didelphis albiventris* (Didelphimorphia, Didelphidae) en su extremo austral, provincia de Rio Negro, Argentina. *Mammalogy Notes* 8, 230. doi:10.47603/mano.v8n1.230

[ref56] Placi G and Di Bitetti M (2006) Environmental situation in the ecoregion of Atlantic Forest of Alto Paraná (Atlantic Forest). *The Environmental Situation in Argentina*, 195–209.

[ref57] Polo-González A, Sánches L and Pacheco V (2019) Helminths of genus *Didelphis* (Didelphimorphia: Didelphidae) or four regions in Perú. *Neotropical Helminthology* 13, 273–286.

[ref58] Portes Santos C, Lent H and Correa Gomes D (1990) The genus *Aspidodera* Railliet and Henry, 1912 (Nematoda: Heterakoidea): Revision, new synonyms and key for species. *Revista Brasileira de Biologia* 50, 1017–1031.

[ref59] Poulin R (2014) Parasite biodiversity revisited: Frontiers and constraints. *International Journal for Parasitology* 44, 581–589. doi:10.1016/j.ijpara.2014.02.003 24607559

[ref60] Ramos DGS, Santos ARGLO, Freitas LC, Correa SHR, Kempe VG, Morgado TO, Aguiar MD, Wolf RW, Rossi RV, Sinkoc AL and Pacheco RC (2016) Endoparasites of wild animals from three biomes in the state of Mato Grosso, Brazil. *Arquivo Brasileiro de Medicina Veterinaria E Zootecnia* 68, 571–578. doi:10.1590/1678-4162-8157

[ref61] Ramos de Souza J, Maldonado A, Jr., Vilela RV, AndradeSilva BE, Barbosa HE, Gomes SR and Thiengo SC (2021) First report of the nematode *Cruzia tentaculata* using molluscs as natural intermediate hosts, based on morphology and genetic markers. *International Journal for Parasitology: Parasites and Wildlife* 15, 105–111. doi:10.1016/j.ijppaw.2021.02.013 33996442 PMC8102712

[ref62] Reiczigel J, Marozzi M, Fábián I and Rózsa L (2019) Biostatistics for parasitologists – A primer to quantitative parasitology. *Trends in Parasitology* 1835, 1–4. 10.1016/j.pt.2019.01.003 30713051

[ref63] Richardson DJ (2006) Life cycle of *Oligacanthorhynchus tortuosa* (Oligacanthorhynchidae), an acanthocephalan of the Virginia opossum (*Didelphis virginiana*). *Comparative Parasitology* 73, 1–6. doi:10.1654/4207.1

[ref64] Richardson DJ and Barnawell EB (1995) Histopathology of *Oligacanthorhynchus tortuosa* (Oligacanthorhynchidae) infection in the Virginia opossum (*Didelphis virginiana*). *Journal of Helminthology* 62, 253–256.

[ref65] Richardson DJ, Gardner SL and Allen JW, Jr (2014) Redescription of *Oligacanthorhynchus microcephalus* (Rudolphi, 1819) Schmidt 1972 (syn. Oligacanthorynchus tortuosa (Leidy, 1850) Schmidt 1972) (Acanthocephala: Oligacanthorhynchidae). *Comparative Parasitology* 81, 53–60. 10.1654/4673.1

[ref66] Sandidge LL (1953) Food and dens of the opossum (*Didelphis virginiana*) in northeastern Kansas. *Transactions of the Kansas Academy of Science* 56, 97. doi:10.2307/3626198

[ref67] Santa Cruz ACM (2006) Ecto y endoparasitosis de *Didelphis albiventris* Temminck, del NEA (Marsupialia, Didelphidae). PhD thesis, Universidad Nacional de la Plata, Argentina.

[ref68] Santa Cruz AMC, Borda JT, Montenegro MA, Gómez LG, Prieto OH and Schebler N (1999) Estudio de ecto y endoparásitos en *Didelphis albiventris* (Comadreja Overa), Marsupialia, Didelphidae. *Comunicaciones Científicas y Tecnológicas*, UNNE. Available at www.unne.edu.ar/cyt/veterinarias/v-025.pdf (accessed 25 July 2015).

[ref69] Scheibel RP, Catzeflis F and Jimenez FA (2014) The relationships of marsupial-dwelling Viannaiidae and description of *Travassostrongylus scheibelorum* sp. n. (Trichostrongylina: Heligmosomoidea) from mouse opossums (Didelphidae) from French Guiana. *Folia Parasitologica* 61, 242–254. doi:10.14411/fp.2014.032 25065130

[ref70] Sikes RS and Gannon WL (2011) Animal Care and use committee of the American Society of Mammalogists. Guidelines of the American Society of Mammalogists for the use of wild mammals in research. *Journal of Mammalogy* 92, 235–253. 10.1093/jmammal/gyw078 PMC5909806 29692469

[ref71] Silva MGQ and Costa HMA (1999) Helminths of white-bellied opossum from Brazil. *Journal of Wildlife Diseases* 35, 371–375. 10231765 10.7589/0090-3558-35.2.371

[ref72] Spasskii AA (1951) Anoplocephalate tapeworms of domestic and wild animals. In *Essentials of Cestodology*. Moscow: The Academy of Sciences of the USSR, 783.

[ref73] Teodoro AKM, Cutolo AA, Motoie G, da Silva Meira-strejevitch C, Pereira-Chioccola VL, Fernandes Mendes TM and Marques Allegretti S (2019) Gastrointestinal, skin and blood parasites in *Didelphis* spp. from urban and sylvatic areas in São Paulo State, Brazil. *Veterinary Parasitology: Regional Studies and Reports* 16, 100286. doi:10.1016/j.vprsr.2019.100286 31027595

[ref74] Travassos L (1914) Trichostrongylideos Brazileiros (III Nota Previa). *Brazilian Journal of Medical and Biological Research* 28, 325–327.

[ref75] Travassos L (1920) Contribuições para o conhecimento da fauna helmintolojica brasileira X. Sobre as especies do gênero *Turgida*. *Memorias Do Instituto Oswaldo Cruz* 12, 73–77.

[ref76] Travassos L (1922) Contribuições para o conhecimento da fauna helmintolojica brasileira XVI: *Cruzia tentaculata* (RUD.,1819). *Memorias Do Instituto Oswaldo Cruz* 14, 88–94. doi:10.1590/s0074-02761922000100004

[ref77] Travassos L (1937) *Revisao da Familia Trichostrongylidae Leiper, 1912*. Rio do Janeiro, Brazil: Monographias do Instituto Oswaldo Cruz.

[ref78] Valente R, Diaz JI, Salomón OD and Navone GT (2016) The role of *Phyllocaulis variegatus* (Mollusca: Veronicellidae) in the transmission of digenean parasites. *Revista Mexicana de Biodiversidad* 87, 255–257.

[ref79] Vicente JJ, Oliveira Rodrigues H, Correa Gomes D and Magalhaes Pinto R (1997) Nematóides do Brasil. Parte V: Nematóides do Mamíferos. *Revista Brasileira de Biologia* 14(1), 1–452. Supl

[ref80] Wilson DE and Reeder DM (2005) (Eds.), *Mammal Species of the World: a Taxonomic and Geographic Reference*. 3rd edn. Baltimore, MD: Johns Hopkins University Press, 2142.

[ref81] Zabott MV, Pinto SB, Viott ADM, Gruchouskei L and de Barros Bittencourt LHF (2017) Helmintofauna de *Didelphis albiventris* (Lund, 1841) no município de Palotina, Paraná, Brasil. *Arquivos de Ciências Veterinárias E Zoologia Da UNIPAR* 20, 19–22. doi:10.25110/arqvet.v20i1.2017.6315

